# Coated cobalamin alleviates heat stress in lactating Holstein cows by enhancing ruminal propionate production, antioxidant capacity, and hepatic mitogen-activated protein kinase signaling

**DOI:** 10.1186/s40104-026-01482-z

**Published:** 2026-09-03

**Authors:** Yongjia Liu, Zitai Guo, Na Cao, Zhicheng Diao, Lu Ma, Yongxin Yang, Yu Zhang, Dengpan Bu

**Affiliations:** 1 State Key Laboratory of Animal Nutrition and Feeding, Institute of Animal Science, Chinese Academy of Agricultural Sciences, No. 2 Yuanmingyuan West Road, Beijing, 100193 People’s Republic of China; 2 College of Food Science and Engineering, Qingdao Agricultural University, Qingdao, 266109 People’s Republic of China; 3 Ningxia Hui Autonomous Region Animal Husbandry Workstation, Yinchuan, 750001 People’s Republic of China; 4 China-Ireland Dairy Sustainable Development Center, Beijing, 100193 People’s Republic of China

**Keywords:** Cobalamin, Heat stress, MAPK signaling pathway, Milk production, Propionate

## Abstract

**Background:**

Heat stress compromises dairy cow productivity and metabolic health through impaired nutrient partitioning and oxidative stress. Cobalamin is an essential cofactor for methylmalonyl-CoA mutase, the key enzyme regulating ruminal propionate production and hepatic gluconeogenic metabolism. However, the underlying molecular mechanisms, particularly the hepatic transcriptional responses to cobalamin under heat stress conditions, remain unclear. This study evaluated the effects of coated cobalamin (CCA) supplementation on lactation performance, nutrient digestibility, rumen fermentation, blood metabolites, and hepatic transcriptome in heat-stressed Holstein cows.

**Results:**

Forty Holstein cows (2.25 ± 0.07 parity; 105 ± 10.4 DIM; 41.5 ± 6.83 kg/d milk yield) were assigned to two groups (*n* = 20) in a randomized block design: (1) control (CON; without CCA) or (2) CCA (addition of CCA at 6 g/d). The experimental period was 64 days, during which the mean temperature-humidity index was 77.1 ± 2.49 (mean ± SD). Respiratory rate was lower in CCA-fed cows than in CON cows. Nutrient intake did not differ, whereas fat-corrected milk, milk protein yields, and feed efficiency were greater for cows receiving CCA. Supplementation with CCA increased ruminal total volatile fatty acid concentration and propionate proportion, but decreased acetate-to-propionate ratio. Apparent digestibilities of organic matter and neutral detergent fiber were greater in the CCA group compared with the CON. Plasma glutathione peroxidase, total antioxidant capacity, and cobalamin were greater, but tumor necrosis factor-α and interleukin-1β were lower for cows in the CCA group. Liver transcriptome analysis revealed that mitogen-activated protein kinase (MAPK), phagosome, and peroxisome proliferator-activated receptor (PPAR) signaling pathways, along with the *JUN*, *FOS*, and *GADD45G* gene family, were key pathways and genes through which CCA regulated hepatic metabolism in heat-stressed cows.

**Conclusion:**

Coated cobalamin addition alleviates heat stress, and increases lactation performance, rumen propionate production, and antioxidant status in dairy cows. Hepatic MAPK signaling pathway mediated the metabolic benefits of CCA in heat-stressed dairy cows. These findings support CCA supplementation as a practical nutritional strategy for mitigating heat stress and provide a mechanistic basis for understanding how targeted nutrient delivery modulates systemic metabolism through hepatic transcriptional regulation.

**Supplementary Information:**

The online version contains supplementary material available at https://doi.org/10.1186/s40104-026-01482-z.

## Introduction

Global climate change has exacerbated the frequency and severity of heat stress in dairy production worldwide [1, 2]. The temperature-humidity index (THI) is widely recognized as a quantitative indicator of heat stress [3]. In China, THI-based milk yield losses per cow reach up to 4 kg/d [1]. Recent meta-analyses indicated that mid- and late-lactation cows were more sensitive to heat stress (THI > 77) than early-lactation cows [4]. In mid-lactation cows, a one-unit increase in THI was associated with a 4.13% decrease in dry matter intake (DMI) and a 3.25% decrease in energy-corrected milk yield [4]. Approximately 50% of the decrease in milk yield was attributed to nutrient redistribution toward thermoregulation and immune activation [5, 6].

Heat stress increases thermoregulatory demands and triggers oxidative stress via the overproduction of reactive oxygen species, thereby reducing the availability of energy and nutrients for mammary gland milk synthesis [5, 7, 8]. Under heat stress, approximately 2 kg/d of glucose was diverted to activated immune and antioxidant systems in cows [6]. Glucose serves as both an energy source and a precursor for lactose synthesis in mammary tissue; it is also essential for leukocyte survival and function [9, 10]. Lactating dairy cows derived 85% to 90% of their glucose requirements from hepatic gluconeogenesis, and approximately 80% of ruminal propionate is absorbed by the liver for glucose synthesis [10, 11]. However, under heat stress conditions, hepatic mRNA expression of the gluconeogenic enzyme phosphoenolpyruvate carboxykinase decreased by 20% in heat-stressed cows [12]. Increasing ruminal propionate production promoted hepatic glucose synthesis and redirect more energy and nutrients toward mammary tissue [13–15].

Cobalamin functions as the cofactor of methylmalonyl-CoA mutase, the key enzyme regulating ruminal propionate production and hepatic gluconeogenic metabolism [16]. In the rumen, cobalamin is involved in the synthesis of microbial DNA, acetyl-CoA, and propionyl-CoA, and constitutes an essential nutrient for *Prevotellaceae* growth [17, 18]. A cross-sectional analysis of 903 dairy cows across 46 farms revealed that blood cobalamin concentration was unchanged as dietary cobalt content increased, indicating that direct cobalamin supplementation might be necessary [19]. Given that approximately 80% of dietary cobalamin is utilized or degraded to inorganic cobalt by ruminal microbes, coated cobalamin (CCA), which exhibits low ruminal degradability (24.84%), was formulated for cows [20, 21]. Previous studies have demonstrated that providing cobalamin to in vitro rumen fluid increased propionate production [18]. Dietary CCA supplementation increased milk production, ruminal total VFA concentration, and molar proportion of propionate in dairy cows [21]. Addition of cobalamin increased the hepatic conversion rate of propionate to glucose in lactating ewes [22]. Additionally, within the one-carbon cycle, cobalamin participates in methionine, glutathione, and taurine synthesis, thereby fulfilling a pivotal role in protein synthesis, antioxidant defense, and immune regulation in dairy cows [16].

Although previous investigations have documented the benefits of cobalamin supplementation for ruminal fermentation and milk production [18, 21], the underlying molecular mechanisms, particularly the hepatic transcriptional responses under heat stress conditions, remain unclear. In addition, no study had systematically investigated how CCA influenced the interplay between ruminal propionate production and hepatic metabolic signaling pathways in heat-stressed dairy cows. Given the critical role of cobalamin in ruminal propionate production, antioxidant function, one-carbon cycle metabolism, and hepatic gluconeogenesis, we hypothesized that, under heat stress conditions, CCA supplementation would improve lactation performance by (1) enhancing ruminal propionate production to support hepatic gluconeogenesis, and (2) activating hepatic stress-responsive signaling pathways (e.g., mitogen-activated protein kinase [MAPK]) to improve antioxidant and immune status. Therefore, this study aimed to investigate the effects of CCA supplementation on lactation performance, nutrient digestibility, rumen fermentation, and blood metabolites, and to elucidate the underlying molecular mechanisms through hepatic transcriptome analysis in Holstein dairy cows under summer heat stress conditions.

## Materials and methods

### Animals, experimental design, and diets

The experiment was conducted from May to August 2025 at Yinshanhu Dairy Farm (Xinzhou, Shanxi Province, China). Approval for the experimental protocol was obtained from the Institutional Animal Care and Use Committee of the Institute of Animal Science, Chinese Academy of Agricultural Sciences (No. IAS2023-56).

Forty Holstein cows (parity = 2.25 ± 0.07; milk yield = 41.5 ± 6.83 kg/d; days in milk = 105 ± 10.4 d) were assigned to one of two treatments: (1) control (CON; basal diet without CCA addition; *n* = 20) or (2) CCA (basal diet plus 6 g/d CCA, providing 12 mg of cobalamin per day; *n* = 20). Cows were blocked by parity, days in milk, and milk yield, and randomly assigned to 2 treatments within each block. Cows underwent a 14-d adaptation period (d −14 to −1), after which data were collected for 50 d (d 1 to 50). Baseline samples were collected on d −14.

The CCA supplement (Shanxi Shannong Guomu Technology Co., Ltd., Taiyuan, China) contained 0.20% cobalamin, 17.50% palm fat, 16.50% calcium stearate, 48.60% silica, and 17.20% hydrogenated coconut oil. The cobalamin in CCA was cyanocobalamin (purity ≥ 98%, synthesized by microbial fermentation). The release rate of cobalamin from CCA, determined using the nylon bag technique with ruminally and duodenally cannulated cows, was 24.84% in the rumen and 70.52% in the intestine. The supplementation level was determined based on Wang et al. [21].

The total mixed ration (TMR, Table 1) was formulated to meet the requirements for mid-lactation dairy cows [23], and contained 0.21 mg Co/kg DM (analyzed by atomic absorption spectrometry; Method 999.11, AOAC International, 2005) [24]. Cows were fed ad libitum twice daily at 0700 and 1500 h, with refusals targeted at approximately 5% of the offered feed. Cows had free access to water and were milked 3 times daily at 0600, 1400, and 2200 h. Before morning feeding, CCA (6 g) mixed with 2 kg TMR was fed individually to cows in the CCA group using headlocks.

**Table 1:** Ingredient and chemical composition of basal diet

Item	Content
Ingredient, % DM	
Corn silage	26.0
Alfalfa hay	13.0
Oat hay	11.0
Corn grain (ground)	24.0
Wheat bran	6.00
Soybean meal	10.6
Rapeseed meal	2.50
Cottonseed meal	5.00
Ca(HCO_3_)_2_	0.60
NaCl	0.50
CaHPO_4_	0.30
Premix^1^	0.50
Chemical composition	
OM, % DM	94.5
CP, % DM	17.2
EE, % DM	3.20
NDF, % DM	31.1
ADF, % DM	19.3
Co, mg/kg DM	0.21
NE_L_, MJ/kg DM	6.63

^1^Contained per kg premix: 20,000 mg Fe, 1,600 mg Cu, 8,000 mg Mn, 7,500 mg Zn, 120 mg I, 60 mg Se, 20 mg Co, 820,000 IU vitamin A, 300,000 IU vitamin D and 10,000 IU vitamin E

**Fig. 1:**
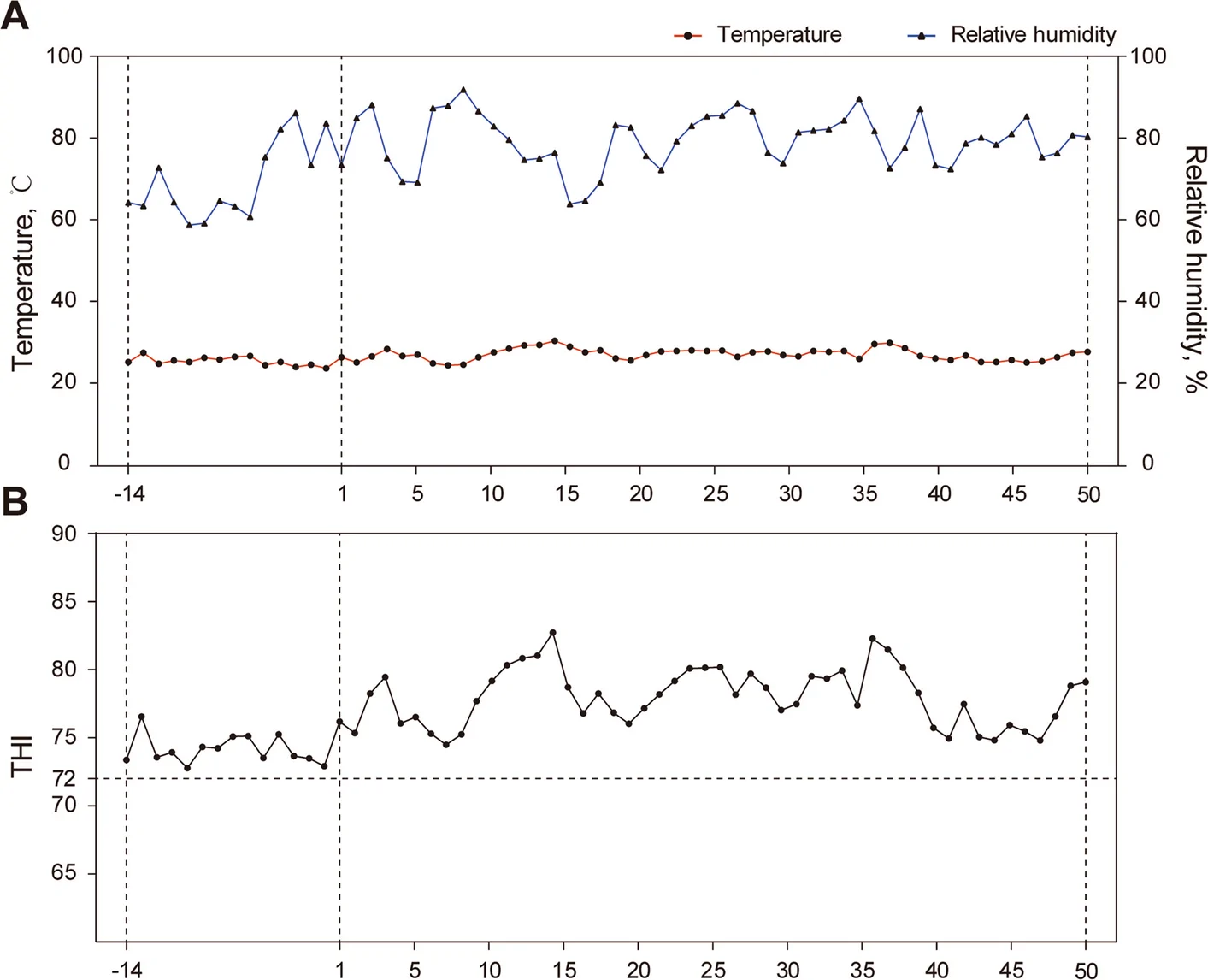
Daily ambient temperature, relative humidity, and temperature-humidity index (THI) during the experimental period. **A** Daily average ambient temperature (●) and relative humidity (▲). **B** Daily THI variation with heat stress threshold THI = 72 (dotted line). Data were recorded at 0530, 1330, and 2100 h daily. THI = (0.81 × T) + (0.99 × T − 14.3) × RH + 46.3, where T = ambient temperature (°C) and RH = relative humidity (proportion)

Cows were housed in the same free-stall barn equipped with cooling systems. Fans (diameter = 1.2 m; wind speed = 2.5 m/s) were activated when ambient temperature exceeded 25 °C. Sprinklers delivered water for 3 min at 10-min intervals when temperature exceeded 28°C. Daily ambient temperature and relative humidity were measured at 0530, 1330, and 2100 h using 5 thermo-hygrometers (Beijing Yaguang Instrument Co., Ltd., Beijing, China) suspended 1.5 m above ground level in the center of the barn. Daily THI values were calculated using the formula: THI = (0.81 × T) + [(0.99 × T − 14.3) × RH] + 46.3, where T = ambient temperature (°C) and RH = relative humidity (proportion, not percentage) [1]. Daily THI values were averaged to construct a THI curve (Fig. 1). The mean THI during the experiment was 77.1 ± 2.49.

### Physiological measurements

Rectal temperature and respiratory rate were measured on d −14, 1, 10, 20, 30, 40 and 50, and at 0830, 1530, and 2100 h using the method described by Wijffels et al. [25]. Measurements were scheduled to avoid feeding and milking times to minimize animal stress and ensure data accuracy. A glass mercury thermometer (Nasco, Fort Atkinson, WI, USA) was inserted 5 cm into the rectum for 2 min to measure rectal temperature. The respiratory rate was determined by counting flank movements per minute. Daily mean values were calculated for each cow.

### Feed intake, milk yield and composition

The TMR offered and the refusals were weighed daily for each cow. Samples of TMR and orts were collected on d −14, 1, 10, 20, 30, 40, and 50, pooled by group, and dried at 65 °C for 48 h to determine DM content (Method 942.05; AOAC International, 2005) [24]. Milk yield was recorded automatically at each milking using a continuous-flow milk meter (FreeFlow 50, Afimilk Ltd., Kibbutz Afikim, Israel). On d −14, 1, 10, 20, 30, 40 and 50, milk samples were collected using an in-line sampler (MMS12, Foss Electric, Hillerød, Denmark) and composited by cow according to morning (40 mL), afternoon (30 mL), and evening (30 mL) milk weights. Analysis of milk components, including true protein, fat, lactose and urea nitrogen, was performed by infrared spectroscopy (MilkoScan FT1, Foss Electric, Hillerød, Denmark). The fat-corrected milk (FCM) yield was calculated according to the formula: FCM (kg/d) = 0.4 × milk yield (kg/d) + 15 × milk fat yield (kg/d) [23].

### Nutrient digestibility

A digestibility trial was conducted from d −17 to −15, and from d 43 to 46. Fecal samples (200 to 400 g) were obtained via direct rectal collection at 0730, 1000, 1600, and 2000 h daily. Samples were immediately placed on ice, pooled by cow to create composite samples (~3 kg), and then a 400-g subsample was stored at −20 °C. TMR samples were collected daily during the feces collection period and stored at −20 °C.

Frozen samples were thawed, dried at 65 °C for 48 h, and ground through a 1-mm screen. Samples were analyzed for DM (Method 934.01), OM, CP (Method 976.05) and ADF (Method 973.18; AOAC International, 2005) [24]. The NDF was determined using heat-stable α-amylase and sodium sulfite [26]. Acid-insoluble ash (AIA) was determined and employed as an internal marker for calculating apparent total-tract digestibility according to Furuichi and Takahashi [27]: Digestibility (%) = {1 − [(AIA in feces/AIA in feed) × (nutrient in feed/nutrient in feces)]} × 100.

### Rumen fermentation

Rumen fluid samples (~150 mL) were collected at 3 h after morning feeding (1000 h) on d −16, −15, 42, and 43 using an oral stomach tube. The first 150 mL was discarded to minimize saliva contamination, and the subsequent 150 mL was retained for analysis. Ruminal pH was measured immediately using a portable pH meter (model 6250, Yibo Instruments Co., Ltd., Shanghai, China). Samples were strained through 4 layers of cheesecloth. For VFA analysis, 5 mL of ruminal fluid was mixed with 1 mL of 25% (w/v) metaphosphoric acid and stored at −20 °C. Concentrations of acetate, propionate, butyrate, valerate, isobutyrate and isovalerate were determined by gas chromatography (GC-2014C, Beijing Beifen-Ruili Analytical Instrument Co., Ltd., Beijing, China) using 2-ethylbutyric acid as the internal standard [28].

### Blood metabolites

Blood samples (10 mL) were collected from the coccygeal vessel into sodium heparin-evacuated tubes (VACUETTE, Greiner Bio-One International GmbH, Kremsmünster, Austria; #456083) at 3 h after morning feeding (1000 h) on d −16, −15, 42, and 43. Samples were centrifuged at 3,000 × *g* and 4 °C for 15 min within 2 h of collection. Plasma was harvested and stored at −20 °C. Analysis of plasma metabolites was performed using colorimetric kits (Nanjing Jiancheng Bioengineering Institute, Nanjing, China) for glucose (#A154-1-1), total protein (#A045-4-1), albumin (#A028-2-1), glutathione peroxidase (GSH-Px; #A005-1-2), nonesterified fatty acids (NEFA; #A042-1-1) and total antioxidant capacity (T-AOC; #A015-1-2). A Hitachi 7600 automated analyzer (Hitachi Co., Tokyo, Japan) was used for most assays, while NEFA and T-AOC were determined with a UV spectrophotometer (Yipu (Shanghai) Instrument Co., Ltd., China). The intra- and inter-assay coefficients of variation were below 3% for glucose, albumin, GSH-Px, NEFA, and T-AOC; below 1.8% for total protein; and below 4.5% for albumin. Plasma beta-hydroxybutyrate (BHB; YJ851236), insulin (INS; YJ669875), tumor necrosis factor alpha (TNFα; YJ265412), interleukin-1β (IL-1β; YJ33174), interleukin-6 (IL-6; YJ612352) and cobalamin (VB_12_; YJ554196) concentrations were determined using commercial ELISA kits (Shanghai Enzyme-linked Biotechnology Co., Ltd., Shanghai, China) following the manufacturer’s protocols, and the intra- and inter-assay CVs were ≤ 15%.

### Liver transcriptome analysis

On the morning of d 50, following milking (0600 h) and prior to feeding (0700 h), 5 cows per group were randomly selected for liver biopsy. The timing was selected to represent the end of the experimental period while ensuring the animals were in a consistent metabolic state (post-milking, pre-feeding). Cows were restrained in headlocks, and the biopsy site (10^th^ intercostal space on the right side) was shaved and disinfected with 70% ethanol, and local anesthesia was induced with 5 mL of 2% procaine hydrochloride. A percutaneous liver biopsy was carried out with a 14-gauge Tru-Cut biopsy needle (CareFusion, San Diego, CA, USA) under ultrasound guidance. Approximately 1.0 g of liver tissue was immediately frozen in liquid nitrogen and kept at −80 °C for RNA extraction.

Total RNA was extracted using TRIzol reagent (Invitrogen, Carlsbad, CA, USA) following the manufacturer's guidelines. RNA quality was assessed using a NanoDrop 2000 spectrophotometer (Thermo Fisher Scientific, Waltham, MA, USA) for concentration and purity, and an Agilent 2100 Bioanalyzer (Agilent Technologies, Santa Clara, CA, USA) for integrity. Samples with an RNA integrity number (RIN) above 7.0 were subjected to library construction.

The NEBNext Ultra RNA Library Prep Kit for Illumina (NEB, Ipswich, MA, USA) was used for RNA sequencing library construction, strictly following the manufacturer's protocol. Briefly, mRNA was enriched using oligo(dT) magnetic beads, fragmented, and reverse-transcribed into cDNA. After end repair, A-tailing, and adapter ligation, cDNA fragments were purified, amplified by PCR, and subsequently quantified using a Qubit 2.0 Fluorometer (Life Technologies, Carlsbad, CA, USA). The constructed libraries were pooled and sequenced on an Illumina NovaSeq 6000 platform (Illumina, San Diego, CA, USA) to generate 150-bp paired-end reads.

Raw reads were filtered to remove adapter sequences and low-quality reads using Trimmomatic (version 0.36; http://www.usadellab.org/cms/?page=trimmomatic). Clean reads were mapped to the bovine reference genome (ARS-UCD1.2) using HISAT2 (version 2.1.0; https://daehwankimlab.github.io/hisat2/). Gene expression levels, expressed as fragments per kilobase of transcript per million mapped reads (FPKM), were quantified using StringTie (version 1.3.4; https://ccb.jhu.edu/software/stringtie/). Differential expression analysis was performed using DESeq2 (version 1.26.0; https://bioconductor.org/packages/release/bioc/html/DESeq2.html) with a threshold of fold change (FC) ≥ 1.5 and an adjusted *P*-value < 0.05 [29]. Although the sample size for liver transcriptome analysis (*n* = 5 per group) was limited, it was consistent with previous ruminant transcriptome studies [30, 31]. The selection of 5 cows per group was based on resource availability and the exploratory nature of the transcriptomic analysis, with subsequent validation recommended in future studies. Kyoto Encyclopedia of Genes and Genomes (KEGG) pathway enrichment analyses were conducted using clusterProfiler (version 3.14.3; https://bioconductor.org/packages/release/bioc/html/clusterProfiler.html) to identify significantly enriched biological processes and pathways.

### Statistical analysis

Data were analyzed using the MIXED procedure of SAS/STAT software, Version 9.4 of the SAS System for Windows (Copyright^©^ 2016 SAS Institute Inc., Cary, NC, USA). For lactation performance, nutrient intake, and feed efficiency, the model included fixed effects of block and treatment, with cow within block × treatment as a random effect. Repeated measures were analyzed using a first-order autoregressive [AR(1)] covariance structure with the Kenward-Roger approximation for denominator degrees of freedom. The appropriateness of the covariance structure was evaluated using the Akaike information criterion (AIC) and the Schwarz Bayesian criterion (BIC). For nutrient digestibility, rumen fermentation, blood metabolites, and liver transcriptome data, the simplified model included fixed effects of block and treatment. Rumen fermentation data were analyzed with sampling day as a fixed effect; when treatment × day interaction was not significant (*P* > 0.05), main effects were presented. Data violating normality or homogeneity assumptions were log-transformed before analysis. Treatment means were separated using the PDIFF option of the LSMEANS statement when the main effect of treatment was significant (*P* < 0.05). Data are presented as least squares means with pooled SEM. Significance was declared at* P* ≤ 0.05, and tendencies were discussed at 0.05 < *P* ≤ 0.10.

## Results

### Physiological indicators and lactation performance

The rectal temperature did not differ between the CCA and the CON groups (*P* = 0.221), whereas respiratory rate was lower (34.8 vs. 41.8 breaths/min; *P* < 0.001) in the CCA group (Table 2). The reduction in respiratory rate in CCA cows was consistently observed from d 10 onward (Fig. S1). The treatment × time interaction had a significant effect on respiratory rate (*P* = 0.007), reflecting a progressively greater reduction in respiratory rate in CCA-fed cows compared with CON cows over the course of the experiment (Fig. S1). The main effect of time was significant for both rectal temperature and respiratory rate (*P* < 0.001), with both parameters increasing from d 1 to d 30 as ambient temperature rose, followed by a slight decline toward d 50.

**Table 2:** Effects of coated cobalamin (CCA) supplementation on physiological indicators and lactation performance in heat-stressed dairy cows (*n* = 20 per treatment)

Item	Treatments^1^		SEM	*P*-value^2^
	CON	CCA		
Rectal temperature, °C	38.4	38.2	0.074	0.221
Respiratory rate, breaths/min	41.8	34.8	0.835	< 0.001
Nutrient intake, kg/d				
DM	27.4	27.3	0.114	0.465
OM	25.4	25.6	0.283	0.695
CP	4.63	4.67	0.051	0.712
NDF	8.37	8.45	0.093	0.699
ADF	5.19	5.24	0.058	0.698
Milk yield, kg/d				
Milk	42.9	44.8	0.424	0.020
FCM^3^	45.1	47.4	0.514	0.036
Fat	1.87	1.96	0.043	0.142
Protein	1.51	1.59	0.017	0.019
Lactose	2.11	2.19	0.022	0.080
Milk composition, %				
Fat	4.35	4.39	0.055	0.759
Protein	3.51	3.54	0.016	0.359
Lactose	4.92	4.89	0.027	0.505
Efficiency, kg/kg				
Milk/DMI	1.56	1.65	0.019	0.030
FCM/DMI	1.65	1.74	0.031	0.036
Milk urea nitrogen, mg/dL	10.3	11.1	0.321	0.244

^1^CON = control (without CCA addition), CCA = 6 g/d of coated cobalamin

^2^Treatment × time interaction not significant for all variables (*P* > 0.10)

^3^FCM = 0.4 × milk yield +15 × fat yield

No significant differences in nutrient intake were observed between groups for DM, OM, CP, NDF, and ADF(*P* > 0.05); however, cows receiving CCA supplementation produced 4.43% more milk (44.8 vs. 42.9 kg/d; *P* = 0.020) and 5.10% more FCM (47.4 vs. 45.1 kg/d; *P* = 0.036) than cows in the CON group (Table 2). Milk protein yield increased by 5.30% (*P* = 0.019) for cows in the CCA group, while milk lactose yield showed only a numerical trend toward a difference between the CCA and CON groups (*P* = 0.080). Milk fat yield, milk component concentrations, and urea nitrogen content did not differ (*P* > 0.05) between the 2 groups. Compared with the CON group, milk/DMI was 5.77% greater (*P* = 0.030) and FCM/DMI was 5.45% greater (*P* = 0.036) in the CCA group. The main effect of time was significant for yields of milk and FCM (*P* < 0.001; Fig. S2).

### Rumen fermentation and nutrient digestibility

Rumen fermentation characteristics and apparent digestibility are reported in Table 3. Ruminal pH was similar (*P* = 0.424) between the CCA and CON groups, whereas total VFA concentration was greater (163 vs. 156 mmol/L; *P* = 0.029) in the CCA group. Acetate concentration increased by 3.03% (98.5 vs. 95.6 mmol/L; *P* = 0.023) in the CCA group compared with the CON group, whereas its molar proportion did not differ significantly (*P* = 0.061) between the 2 groups. Both the concentration (*P* = 0.002) and the molar proportion of propionate (*P* = 0.047) were greater in the CCA group compared with the CON group, resulting in a lower acetate-to-propionate ratio (2.49 vs. 2.70; *P* = 0.022) in the CCA group. Concentrations and molar proportions of butyrate, valerate, isobutyrate, and isovalerate did not differ (*P* > 0.05) between the 2 groups.

**Table 3:** Effects of coated cobalamin (CCA) supplementation on rumen fermentation characteristics and apparent total-tract digestibility in heat-stressed dairy cows (*n* = 20 per treatment)

Item	Treatments^1^		SEM	*P*-value
	CON	CCA		
Ruminal pH	6.18	6.22	0.029	0.424
VFA concentration, mmol/L				
Total VFA	156	163	1.398	0.029
Acetate	95.6	98.5	0.634	0.023
Propionate	35.5	39.6	0.471	0.002
Butyrate	18.0	18.3	0.120	0.215
Valerate	2.69	2.61	0.032	0.232
Isobutyrate	1.65	1.68	0.030	0.638
Isovalerate	2.26	2.29	0.029	0.657
VFA, mol/100 mol				
Acetate	61.4	60.4	0.256	0.061
Propionate	22.8	24.3	0.220	0.047
Butyrate	11.6	11.2	0.117	0.153
Valerate	1.73	1.60	0.024	0.068
Isobutyrate	1.06	1.03	0.019	0.467
Isovalerate	1.45	1.41	0.022	0.273
Acetate:Propionate	2.70	2.49	0.056	0.022
Apparent digestibility, %				
DM	70.9	72.0	0.498	0.272
OM	72.8	74.9	0.450	0.021
CP	73.3	75.2	0.734	0.209
NDF	50.0	53.8	0.752	0.009
ADF	44.1	42.4	0.497	0.077

^1^CON = control (without CCA addition), CCA = 6 g/d of coated cobalamin

Apparent total-tract digestibility of DM did not differ (*P* = 0.272) between groups, whereas OM digestibility was greater (74.9% vs. 72.8%; *P* = 0.021) in CCA cows. Although CP (*P* = 0.209) and ADF (*P* = 0.077) digestibility did not differ, NDF digestibility increased (53.8% vs. 50.0%; *P* = 0.009) in the CCA group.

### Blood metabolites

Plasma concentrations of glucose, INS, total protein, albumin, NEFA, BHB and IL-6 did not differ (*P* > 0.05) between the CCA and CON groups (Table 4). However, GSH-Px activity was 7.65% greater (*P* = 0.014) and T-AOC was 18.1% greater (*P* = 0.011) in the CCA group than in the CON group. The TNFα concentration was 16.0% lower (*P* = 0.016) and IL-1β concentration was 7.42% lower (*P* = 0.025) in the CCA group than in the CON group. Additionally, plasma cobalamin concentration was 13.6% greater (*P* = 0.021) in the CCA group than in the CON group.

**Table 4:** Effects of coated cobalamin (CCA) supplementation on plasma metabolites and antioxidant parameters in heat-stressed dairy cows (*n* = 20 per treatment)

Item	Treatments^1^		SEM	*P*-value
	CON	CCA		
Cobalamin, pmol/L	236	268	8.451	0.021
Glucose, mmol/L	3.19	3.27	0.052	0.459
INS, mU/L	19.6	22.3	0.890	0.140
Total protein, g/L	72.2	75.6	1.173	0.161
Albumin, g/L	38.3	40.5	0.820	0.185
NEFA, μmol/L	364	378	9.951	0.501
BHB, μmol/L	424	442	13.54	0.523
GSH-Px, U/mL	405	436	6.356	0.014
T-AOC, U/mL	3.26	3.85	0.075	0.011
TNFα, pg/mL	257	216	8.494	0.016
IL-1β, pg/mL	337	312	5.450	0.025
IL-6, pg/mL	286	274	3.433	0.098

^1^CON = control (without CCA addition); CCA = 6 g/d of coated cobalamin

^2^*INS* Insulin, *NEFA* Non-esterified fatty acids, *BHB* Beta-hydroxybutyrate, *GSH-Px* Glutathione peroxidase, *T-AOC* Total antioxidant capacity, *TNFα* Tumor necrosis factor alpha, *IL-1β* Interleukin-1 beta, *IL-6* Interleukin-6

### Liver transcriptome

**Fig. 2:**
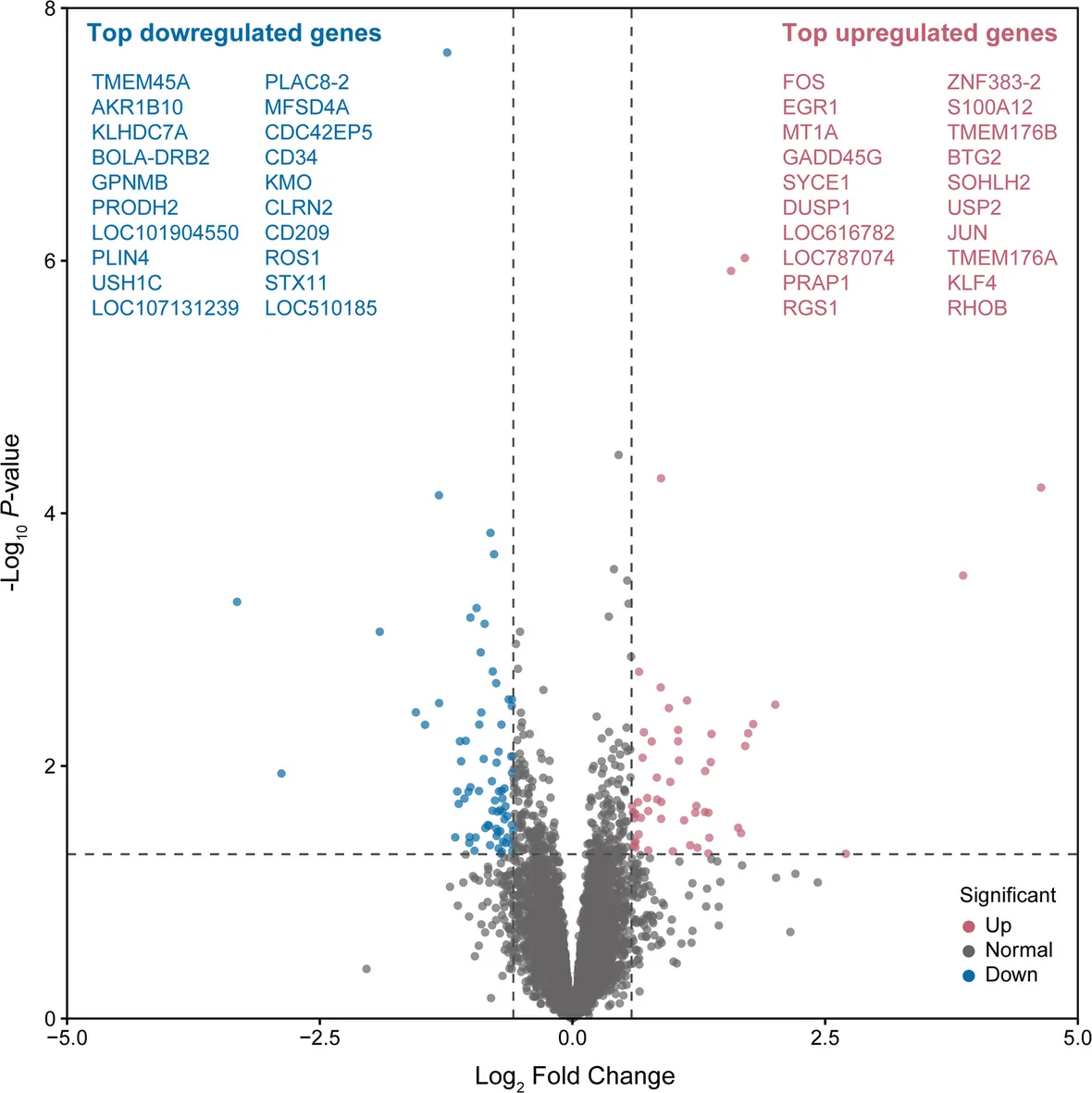
Volcano plot of DEGs in liver tissue of Holstein cows harvested on d 50 after exposure to summer heat stress without CCA (CON, *n* = 5) or with CCA 6 g/d addition (CCA, *n* = 5). The *y*-axis displays the −log_10_ *P*-value for each gene, and the *x*-axis displays the log_2_ fold change of that gene relative to the CON group. Pink dots indicate upregulation, blue dots indicate downregulation in the CCA group (−log_10_ *P*-value > 1.3; *P* < 0.05), indicated by a horizontal dotted line; |log_2_FC| ≥ 0.585 (|FC| ≥ 1.5), indicated by vertical dotted lines, and gray dots indicate nonsignificance. Insets display the top 20 upregulated (pink) and downregulated (blue) genes in CCA relative to CON cows based on log_2_ fold change

A total of 12,509 genes were identified in liver tissue (Table S2). Using a *P*-value cut-off of 0.05 and FC ≥ 1.5, 127 differentially expressed genes (DEGs) were identified between the CCA and CON groups, comprising 55 upregulated and 72 downregulated genes in CCA cows (Table S3). The magnitude of transcriptional changes ranged from log_2_FC = −3.32 to 4.64 (Fig. 2). Notable upregulated genes included immediate-early response genes (*FOS,* log_2_FC = 4.64; *EGR1*, log_2_FC = 3.87) and stress-responsive genes (*MT1A*, log_2_FC = 2.70; *GADD45G*, log_2_FC = 2.01), whereas downregulated genes comprised metabolic genes (*TMEM45A*, log_2_FC = −3.32; *AKR1B10,* log_2_FC = −2.88).

**Fig. 3:**
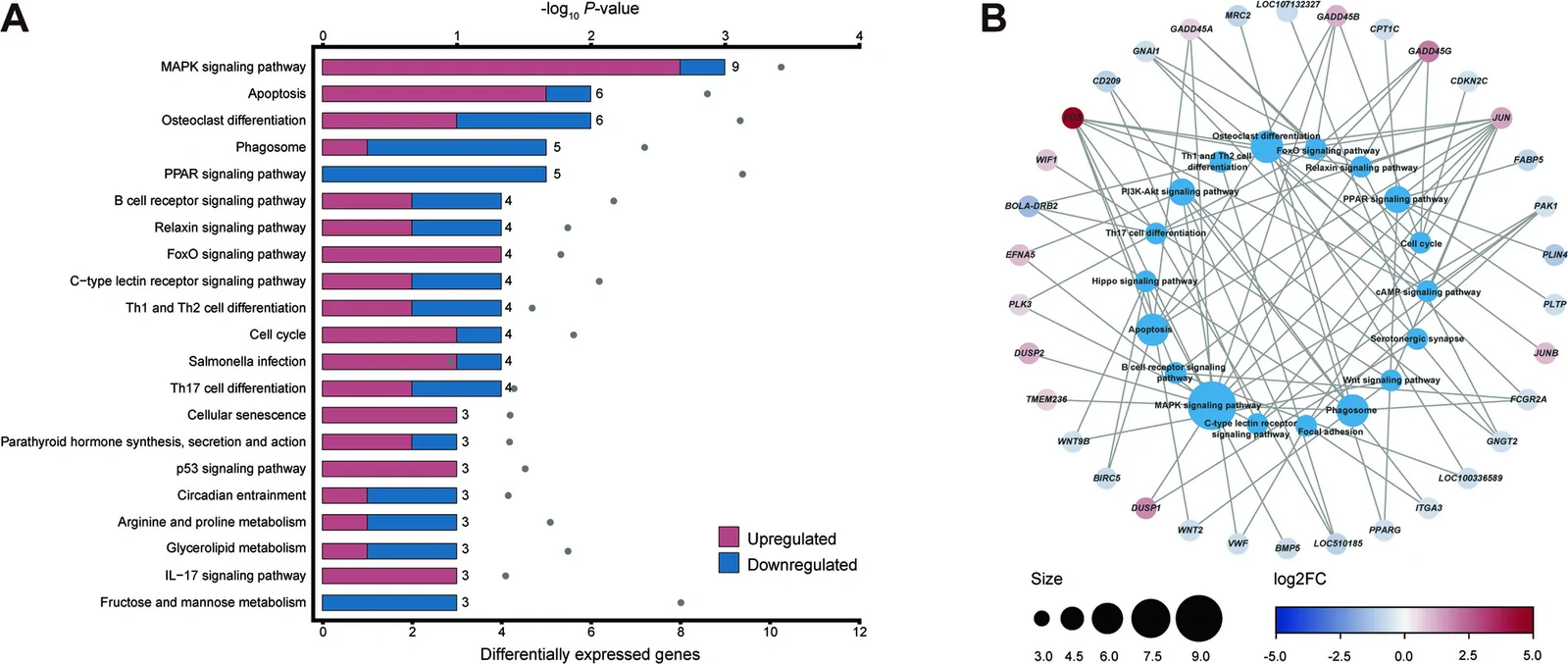
Enriched KEGG pathways and differentially expressed genes (DEGs) in liver tissue of Holstein cows harvested on d 50 after exposure to summer heat stress without CCA (CON, *n* = 5) or with CCA 6 g/d addition (CCA, *n* = 5). **A** Significantly enriched KEGG pathways (*P* < 0.05). The *y*-axis displays the pathway name. The *x*-axis displays the number of DEG within each pathway and the significance of enrichment (−log_10_ *P*-value, black dots). Pink bars display the number of upregulated genes, and blue bars display the number of downregulated genes in each pathway in CCA to CON cows. **B** Network topology of pathway and DEG cross-talk (DEG count ≥ 4). Inner circular nodes: KEGG pathways (node size proportional to DEG count). Outer circular nodes: hub genes (node color indicates log_2_ fold change: Red = upregulated, blue = downregulated; color intensity proportional to |log_2_FC|). Connecting lines indicate gene-pathway associations

KEGG analysis identified 45 pathways enriched with at least 3 DEGs (Table S4). Using a *P*-value cut-off of 0.05, 22 pathways were identified (Fig. 3A), and the top 5 pathways (DEGs ≥ 4) included MAPK signaling, peroxisome proliferator-activated receptor (PPAR) signaling pathways, osteoclast differentiation, apoptosis and phagosome. Among these, the osteoclast differentiation pathway was not discussed in the following sections.

Network analysis identified 6 hub genes connecting more than 4 pathways: *JUN*, *FOS*, *GADD45A*, *GADD45B*, *GADD45G*, and *PAK1*, among which *JUN*, *FOS*, and *GADD45G* ranked within the top 20 upregulated genes (Fig. 3B). The MAPK signaling pathway was enriched with 9 DEGs, of which 8 were upregulated (*JUN*, *GADD45G*, *GADD45B*, *GADD45A*, *FOS*, *EFNA5, DUSP2*, *DUSP1*) and 1 was downregulated (*PAK1*). The apoptosis pathway contained 6 DEGs, with 5 upregulated (*FOS*, *GADD45A*, *GADD45B*, *GADD45G*, *JUN*) and 1 downregulated (*BIRC5*). The phagosome pathway was enriched with 5 DEGs (1 upregulated, 4 downregulated), including upregulated *TMEM236* and downregulated *BOLA-DRB2*, *CD209*, *FCGR2A,* and *MRC2*. The PPAR signaling pathway was characterized by downregulated *CPT1C*, *FABP5*, *PLIN4*, *PLTP,* and *PPARG*.

## Discussion

The present study supported our hypothesis. Dietary CCA supplementation alleviated heat stress in lactating Holstein cows, as evidenced by reduced respiratory rate and increased milk yield, feed efficiency, and antioxidant capacity. The integrated analysis of lactation performance, nutrient digestibility, rumen fermentation, blood metabolites, and hepatic transcriptome revealed a mechanistic pathway linking ruminal propionate production to hepatic metabolic reprogramming through MAPK signaling pathway activation. The following discussion integrated these findings into a cohesive narrative that followed the effects of CCA from ingestion through digestion and absorption to systemic metabolism and, ultimately, milk synthesis.

### Heat stress confirmation and physiological response

The treatment × time interaction for respiratory rate reflected a progressively divergent response between treatments as heat stress intensified during mid-summer (Fig. 1B). The CCA group exhibited a sustained reduction in respiratory rate from d 10 onward, whereas CON cows maintained elevated respiratory rates throughout the experimental period. This divergence likely arose from the cumulative benefits of CCA on ruminal propionate production and systemic antioxidant capacity, which required approximately 10 d to manifest physiologically. The significant time effects for rectal temperature and respiratory rate paralleled the seasonal THI trajectory (Fig. 1B), with both parameters peaking around d 20 to 30 when THI reached its maximum (THI > 80), confirming that cows experienced escalating heat stress during mid-summer. Similarly, the time effects on milk and FCM yields (Fig. S2) reflected the well-documented seasonal decline in lactation performance under heat stress, with yields decreasing as THI increased and partially recovering toward late summer as THI moderated. The mean THI of 77.1 ± 2.49 recorded throughout the experimental period exceeded the established threshold of 72 [1, 3], confirming that cows experienced moderate heat stress. A positive correlation between rectal temperature and respiratory rate has been consistently observed in dairy cattle, with Li et al. [32] reporting an increase in 0.1 °C in rectal temperature for every 4.8 breaths/min elevation in respiratory rate. In CCA-supplemented cows, respiratory rate decreased by 7.0 breaths/min, accompanied by a 0.2 °C reduction in rectal temperature, indicating amelioration of heat stress. This physiological improvement demonstrated that CCA supplementation lessened the thermoregulatory burden of heat exposure, establishing the foundation for subsequent metabolic effects.

### Rumen fermentation and nutrient digestibility

Ruminal pH values of 6.18 and 6.22 in the CON and CCA groups, respectively, fell within ranges considered optimal for microbial proliferation and nutrient breakdown [33]. Although nutrient intake remained unchanged, CCA supplementation increased ruminal total VFA concentration and propionate molar proportion while reducing the acetate-to-propionate ratio. These observations aligned with increased total-tract digestibility of OM and NDF, suggesting that CCA supplementation shifted ruminal fermentation pattern toward increased propionate production. The supplementary CCA released 24.8% of its cobalamin in the rumen. Cobalamin serves as an essential cofactor for methylmalonyl-CoA mutase, which catalyzes the conversion of methylmalonyl-CoA to succinyl-CoA in the propionate production pathway [17]. The increase in ruminal propionate production alleviated the critical metabolic constraint imposed by impaired glucose supply in heat-stressed dairy cows, representing the central initial step in the mechanistic cascade through which CCA exerted its effects.

### Systemic metabolic effects

The elevation of plasma cobalamin concentration was in line with the results of a previous study [21], showing that supplementary cobalamin was absorbed in CCA-supplemented cows. The supplementary CCA released 70.5% of its cobalamin in the intestine. The increase in ruminal propionate concentration in cows receiving CCA did not change plasma glucose concentration but numerically increased lactose yield, indicating that glucose flow to the mammary gland was enhanced. Blood glucose homeostasis was maintained through the coordinated regulation of hepatic gluconeogenesis and peripheral glucose utilization [11]. Unaltered plasma NEFA and BHB levels confirmed metabolic stability and no exacerbation of lipolysis in cows receiving CCA, indicating that increased milk yield was associated with enhanced nutrient digestion and systemic redistribution of absorbed nutrients.

CCA supplementation increased plasma GSH-Px and T-AOC activities while decreasing TNFα and IL-1β concentrations, which was related to the role of cobalamin in glutathione synthesis via the one-carbon cycle [16]. The improvement in antioxidant and immune status in cows receiving CCA likely facilitated the redirection of absorbed nutrients toward mammary tissue [13, 14]. Therefore, by reducing immune system activation, CCA supplementation decreased glucose consumption by activated immune cells during heat stress, allowing more glucose to be available for milk synthesis; this complemented the propionate supply mechanism.

### Hepatic transcriptional reprogramming

The liver transcriptome analysis identified 127 DEGs, and 22 enriched pathways, indicating that CCA supplementation triggered coordinated transcriptional responses in cows under heat stress conditions. The MAPK, apoptosis, phagosome and PPAR signaling pathways, along with DEGs enriched within these pathways, were discussed below.

The MAPK pathway regulates cell proliferation, inflammation, antioxidant defense, and lipid metabolism [34]. The MAPK family comprises three branches: ERK, p38, and JNK [34]. Within the JNK branch, GADD45G, GADD45B, and GADD45A function as upstream activators; JUN and FOS serve as the downstream transcription factors; and DUSP1 and DUSP2 act as negative feedback regulators that prevent excessive activation and consequent oxidative stress or apoptosis [34–36]. The concurrent upregulation of *GADD45* family members, *JUN*, *FOS*, *DUSP1,* and *DUSP2* indicated that balanced JNK/MAPK signaling was activated following CCA addition in heat-stressed cows, consistent with the observed changes in blood GSH-Px, T-AOC, TNFα and IL-1β concentrations. Corroborating these findings, Laranjeira et al. [37] recently reported that vitamin B_12_ supplementation increased MAPK pathway activity through SAM-dependent histone methylation. This raised the possibility that CCA modulated hepatic MAPK signaling via a similar epigenetic mechanism. Studies in monogastric animals have demonstrated that activation of the MAPK signaling pathway alleviates heat stress-induced intestinal damage in broilers [38]. Likewise, greater hepatic MAPK signaling pathway activity and blood GSH-Px and T-AOC activities were observed in stress-resistant compared to stress-sensitive pigs exposed to a plateau environment [39].

Apoptosis is a tightly regulated process of programmed cell death that maintains tissue homeostasis in the liver [40]. In the liver of CCA-supplemented cows, 6 DEGs were enriched in the apoptosis pathway. Downregulated *BIRC5* belongs to the anti-apoptotic gene family, whereas upregulated *FOS*, *JUN,* and *GADD45* family genes are positioned upstream of pro-apoptotic genes [40]. These expression patterns indicated that apoptosis pathway activity remained unaffected, with cell proliferation and death rates maintaining homeostatic balance in cows with CCA addition. Similarly, Zhai et al. [41] observed that the hepatic apoptosis pathway was not affected by heat stress in calves.

The phagosome pathway regulates phagocyte function: phagocytes recognize targets via surface receptors, process internalized targets, and present antigens to T cells, inducing the production of pro-inflammatory cytokines and chemokines [42]. Five DEGs were enriched in this pathway: *TMEM236*, *MRC2* and *CD209* are members of the C-type lectin receptor family; *FCGR2A* belongs to the Fc receptor family, and *BOLA-DRB2* is a component of the classical MHC-II molecule responsible for antigen presentation [43, 44]. Studies have reported that reduced Fc receptor expression alleviated chronic inflammation [43], and *CD209* expression levels directly correlated with phagocytic capacity [45]. Therefore, the downregulation of *MRC2*, *CD209*, *FCGR2A,* and *BOLA-DRB2* was consistent with the decrease in blood TNFα and IL-1β concentrations, indicating suppression of hepatic phagosome activity in CCA-supplemented cows.

In the PPAR pathway, PPARG is activated by fatty acids, whereas FABP5 functions as a lipid ligand chaperone that delivers fatty acids to PPARG in the nucleus [46, 47]. *PLIN4*, *CPT1C*, and *PLTP* are downstream genes of *PPARG* that regulate lipid droplet binding and degradation, mitochondrial fatty acid oxidation, and phospholipid transfer and cholesterol metabolism, respectively [48, 49]. *PLIN4* expression is highly dependent on PPARG activity [50]. The downregulation of *FABP5*, *PPARG*, *PLIN4*, *CPT1C,* and *PLTP* indicated that the activity of the PPAR pathway was suppressed in CCA-supplemented cows.

The balanced activation of the MAPK signaling pathway, characterized by upregulation of *JUN*, *FOS*, *GADD45* family, and *DUSP* genes, enhanced antioxidant defense and inflammatory modulation. The downregulation of phagosome pathway-related genes (*MRC2*, *CD209*, *FCGR2A,* and *BOLA-DRB2*) alleviated hepatic inflammation. The suppression of PPAR signaling (*FABP5*, *PPARG*, *PLIN4*, *CPT1C*, and *PLTP*) reduced hepatic lipid retention and facilitated fatty acid partitioning toward the mammary gland. This coordinated transcriptional response converted the increased ruminal propionate into enhanced systemic antioxidant capacity and milk synthesis in cows receiving CCA.

### Comparison with previous research

The results of this study confirmed and extended previous findings regarding cobalamin. Wang et al. [21] reported that dietary CCA supplementation increased milk yield, ruminal propionate concentration and total-tract nutrients digestibility in dairy cows. Liu et al. [18] demonstrated that supplementing cobalamin to in vitro rumen fluid increased *Prevotella* abundance and propionate production. Others reported that intramuscular cobalamin injection did not alter plasma glucose, NEFA, and BHB concentrations in dairy cows [51], and no correlation was observed between blood cobalamin concentration and NEFA or BHB concentrations during the transition period [52]. The present study confirmed these metabolic findings in heat-stressed dairy cows and provided novel insights that activation of the hepatic MAPK signaling pathway mediated the systemic benefits of CCA.

### Limitation and implications

This study has several limitations that should be acknowledged. First, the sample size for hepatic transcriptome analysis (*n* = 5 per group) was relatively small, reflecting the exploratory nature of this study. Future research should employ Western blotting or immunohistochemistry to corroborate transcriptional changes at the protein level. Second, this study was conducted during a single summer; validation across multiple years and locations is necessary to strengthen generalizability. Third, an uncoated cobalamin treatment group was not included in the experimental design. Inclusion of such a group would have enabled a direct comparison within the study and provided a stronger basis for evaluating the effects of the CCA treatment.

At a supplementation level of 6 g/d, CCA is a commercially available product that can be readily incorporated into the TMR. The observed increases in milk yield (1.9 kg/d) and feed efficiency (5.8%), along with the reduction in respiratory rate (7.0 breaths/min), translate into improved economic benefits and animal welfare. The mechanistic framework, which traces the pathway from ruminal propionate enhancement to hepatic MAPK activation, provides a basis for understanding how cobalamin supports glucose homeostasis and mitigates heat stress responses. Subsequent investigations should focus on validating this pathway and evaluating its relevance under other stress conditions, such as disease challenge or high-production demands.

## Conclusions

Dietary supplementation with CCA at 6 g/d decreased respiratory rate, increased milk yield, and improved feed efficiency in Holstein cows under moderate heat stress conditions. These productive benefits were attributed to a coordinated, multi-tissue mechanism of action. Specifically, the provision of CCA enhanced total VFA production, and shifted fermentation patterns toward propionate generation. In circulation, CCA provision elevated antioxidant capacity (GSH-Px, T-AOC) while reducing the concentrations of pro-inflammatory cytokines (TNFα, IL-1β). At the hepatic level, CCA induced transcriptional reprogramming characterized by balanced activation of the MAPK signaling pathway, which was mediated by *JUN*, *FOS*, and *GADD45G* family genes, alongside suppression of phagosome pathway activity, and downregulation of PPAR signaling. This integrated physiological response alleviated the metabolic burden imposed by heat stress, improved nutrient utilization efficiency, and supported enhanced milk synthesis. These findings support CCA as a practical nutritional strategy for mitigating heat stress in dairy cows and provide a mechanistic basis for understanding how targeted nutrient delivery modulates systemic metabolism through hepatic transcriptional regulation.

## Supplementary Information

Additional file 1: Table S1. Summary statistics of RNA-seq data.

Additional file 2: Table S2. List of all 12,509 genes identified in liver tissue with normalized expression values.

Additional file 3: Table S3. Complete list of 127 DEGs identified in liver transcriptome analysis of heat-stressed Holstein cows supplemented with CCA, including gene symbols, base mean, *P*-value, log_2_FC and FDR.

Additional file 4: Table S4. KEGG pathway enrichment analysis results for upregulated and downregulated genes, including pathway IDs, descriptions, gene counts, and enrichment statistics.

Additional file 5: Fig. S1. Dynamics of respiratory rateand rectal temperaturein heat-stressed dairy cows throughout the experimental period.

Additional file 6: Fig. S2. Dynamics of milk yieldin heat-stressed dairy cows throughout the experimental period, comparing CON and CCA groups.

## Data Availability

All the data generated or analysed for this study are included in this paper. The RNA sequencing data have been deposited in the National Center for Biotechnology Information (NCBI) Sequence Read Archive (SRA) under accession number PRJNA1444757.

## Abbreviations

AIA: Acid-insoluble ash
BHB: Beta-hydroxybutyrate
CCA: Coated cobalamin
CON: Control
DEG: Differentially expressed gene
DMI: Dry matter intake
FCM: Fat-corrected milk
FC: Fold change
GSH-Px: Glutathione peroxidase
IL: Interleukin
INS: Insulin
KEGG: Kyoto Encyclopedia of Genes and Genomes
MAPK: Mitogen-activated protein kinase
NEFA: Non-esterified fatty acids
PPAR: Peroxisome proliferator-activated receptor
T-AOC: Total antioxidant capacity
THI: Temperature-humidity index
TNFα: Tumor necrosis factor alpha
VFA: Volatile fatty acid
